# NaCl pretreatment attenuates *H.pylori*-induced DNA damage and exacerbates proliferation of gastric epithelial cells (GES-1)

**DOI:** 10.1186/s13027-015-0003-3

**Published:** 2015-03-01

**Authors:** Ying Xu, Ying Yan, Ming-xiao Hou, Yun-en Liu

**Affiliations:** Radiation oncology Department of General Hospital of Shenyang Military Command, Shenyang, l10016 China; Emergency Medicine Department of General Hospital of Shenyang Military Command, Laboratory of Rescue Center of Severe Wound and Trauma PLA, 83 Wenhua Road, Shenhe District, Shenyang, l10016 China

**Keywords:** NaCl-pretreatment, *H. pylori*, Gastric epithelial cells, Apoptosis, Proliferation

## Abstract

**Background:**

Both *H. pylori* infection and high salt (NaCl) diet are risks of gastric cancer, however, the interaction pattern of the two is not very clear. Our objective was to investigate the effects of NaCl-pretreated *H. pylori* on DNA damage and proliferation of gastric epithelial cell (GES-1).

**Methods:**

GES-1 cells were co-cultured with *H.pylori* or NaCl-pretreated *H. pylori* (with 30% NaCl) for 24 h. The morphological changes of all cells were observed by inverted phase contrast microscopy and transmission electron microscopy. Oxidative DNA damage was examined by immunofluorescence. Alterations in mitochondrial membrane potential and apoptosis rate were detected by flow cytometry and western blot, and expression of Ki-67, PCNA and P21 were evaluated using the immunocytochemical staining.

**Results:**

GES-1 cells co-cultured with NaCl-pretreated *H.pylori* exhibited morphological changes and oxidative DNA damage. Although no significant disruption of the mitochondrial membrane potential (*ΔΨ*m) and apoptotic rate were observed compared with control groups, there were significant decreased in Bax and Caspase3 proteins and increased in Bcl-2 protein in GES-1 cells infected with *H. pylori*^30^ when compared with GES-1 cells cultured with *H. pylori.* In addition, we found a proliferative effect on GES-1 cells with an increased expression of Ki-67 and PCNA as well as a decreased p21 expression, through which the cells may acquire the potential for malignant transformation.

**Conclusion:**

NaCl-pretreated *H. pylori* possessed the ability to cause cell injury and promote proliferation in gastric epithelial cells.

## Background

Epidemiological evidence suggested a positive association between high salt or and *H. pylori* infection in gastric diseases [1]. Some contaminated foods or water, including those with very high NaCl concentrations may serve as reservoirs for the transmission of *H.pylori* [2]. *H. pylori* has to conquer a tumultuous environment before colonizing the gastric mucosa to cause consequent gastroduodenal diseases. Thus, it is important to investigate the survivability and self-regulation of *H.pylori* exposed to high salt concentrations. In our preliminary studies, we found that *H. pylori* can tolerate salt concentrations and correspondingly change biological characteristics to survive. However, it has never been shown whether *H.pylori* pretreated with high salt concentrations will retain the ability to cause cell oxidative damage and how its effect on proliferation of the gastric epithelial cells changes *in vitro*.

Among the pathogenic mechanisms relevant to gastric carcinogenesis and correlated with *H. pylori* infection, it has been demonstrated that the production of reactive oxygen species (ROS) and subsequent damage to DNA may be quite important. 8-hydroxy-2’-deoxyguanosine (8-OHdG) is a specific product of DNA oxidative damage and commonly recognized as a biomarker of endogenous and exogenous oxidative DNA damage [3,4]. *H. pylori*-associated inflammation is indicated by increased levels of oxidative DNA damage, and increased occurrences of apoptosis and proliferation, which seems to provide the mechanistic link between *H. pylori* infection and gastric carcinogenesis [5]. There is ample evidence that ROS can induce cell proliferation, apoptosis and, at high doses, necrotic cell death. Oxidative DNA damage of cells can lead to mitochondrial transmembrane potential (*ΔΨ*m) collapse [6] which is considered as an initial and irreversible step towards apoptosis [7]. In addition, other studies have demonstrated that *H. pylori* plays a critical role in the evolution of gastritis to gastric carcinoma. During this process, increased proliferation of gastric epithelial cells due to *H. pylori* infection has been observed [8-10]. Ki67 antigen is an important biomarker for the assessment of tumor cell proliferation [11]. PCNA is a major biological index of cell proliferation, which can objectively reflect the proliferation of tumor cells [12]. The p21 protein is a cyclin-dependent kinase(cdk) inhibitory protein that functions as a cell cycle regulator to block the transition from G1 phase to S phase, thus suppressing cell proliferation [13]. *H. pylori* infection may be an initiating step in gastric carcinogenesis through promoting proliferation of gastric epithelial cells along with changes in proliferation-related proteins.

The aim of this study was to identify the ability of *H. pylori* pretreated with high salt to cause cell oxidative damage and describe its biological effects on the proliferation of GES-1 cells.

## Results

### Morphological changes in GES-1 cells infected with *H. pylori*

**Figure 1:**
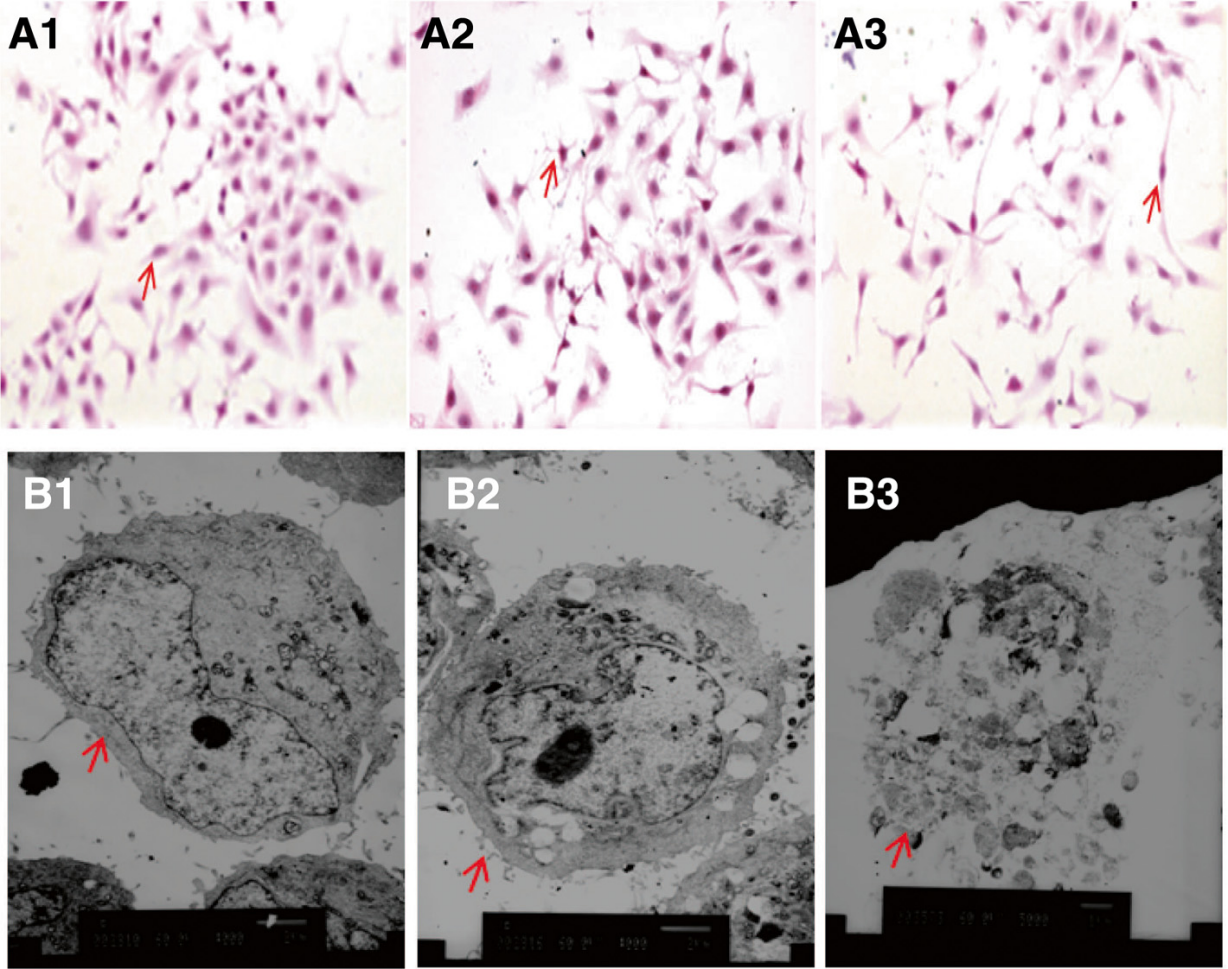
**The effect of** ***H. pylori*** ^***30***^ **on GES-1 cell morphology.** Control GES-1 cells **(A1, B1)**; GES-1 cells co-cultured with *H.pylori* ^*30*^ **(A2, B2)**; GES-1 cells co-cultured with *H. pylori* **(A3, B3)** (HE **×** 200; TEM **×** 4000).

GES-1 control cells presented with a polygonal or fusiform shape, a regular appearance and clear edge (Figure 1A1). After 24 h co-culture with *H.pylori* and *H.pylori*^*30*^ (*H.pylori* pretreated with 30% NaCl)*,* GES-1 cells transformed from multiangular to round or irregular shapes of various sizes, with disrupted cell walls and cytoplasmic leakage(Figure 1A2-A3). Transmission electron microscopy revealed that the GES-1 cells co-cultured with *H. pylori*^*30*^ and *H. pylori* for 24 h were characterized by the loss of microvilli, karyorrhexis and vacuolization of the cytoplasm (Figure 1B1-3).

### NaCl pretreatment attenuated *H. pylori*- induced oxidative DNA damage

**Figure 2:**
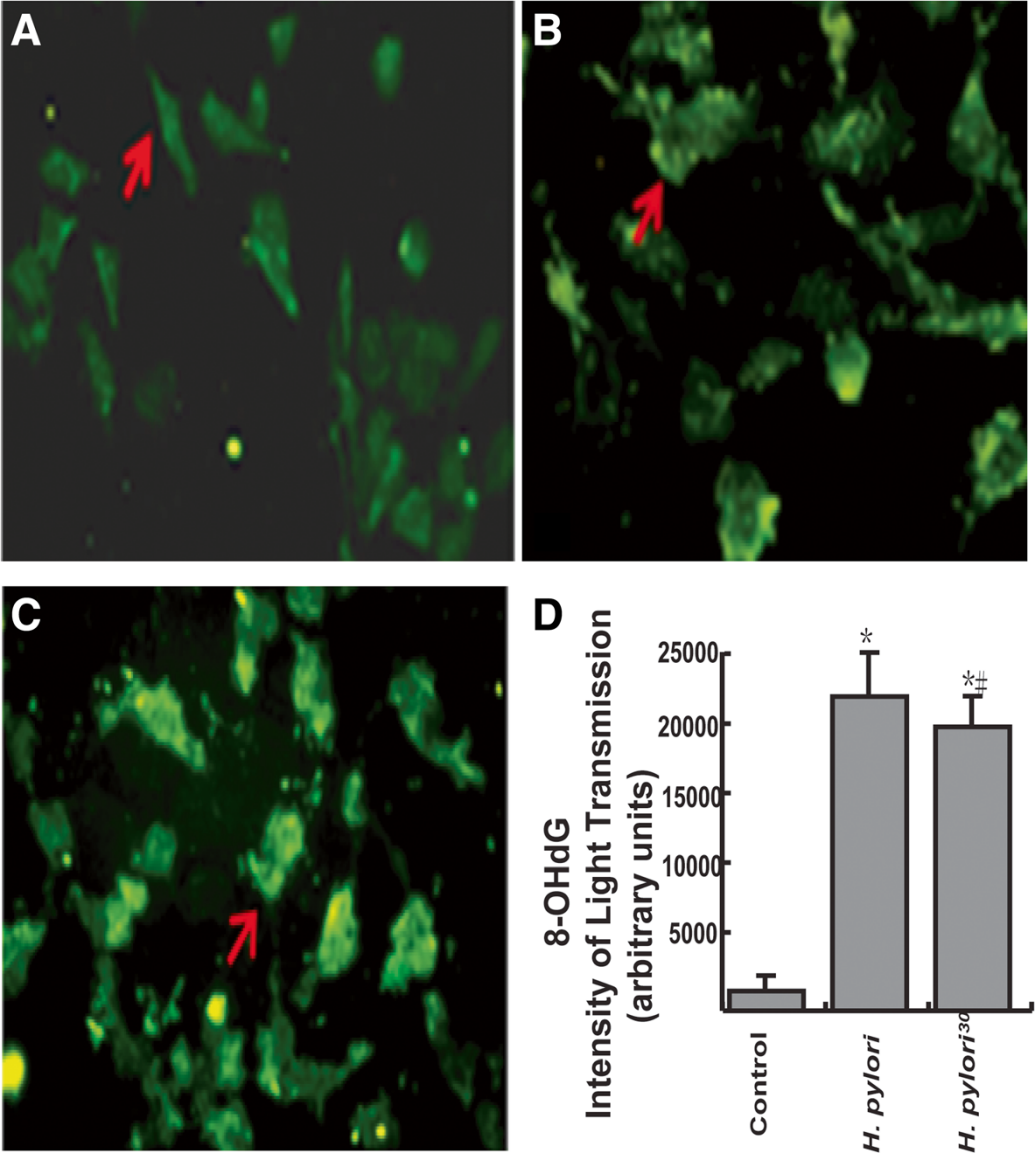
**The expression of 8-OHdG in GES-1 cells detected by immunofluorescence. A**: GES-1 cells (negative control group), cell membrane is distinct and slightly stained; **B**: GES-1 cells co-cultured with *H. pylori*, cell membrane, cytoplasm and nucleus are all strongly stained; **C**: GES-1 cells co-cultured with *H. pylori* ^*30*^, cell membrane, cytoplasm and nucleus are all stained (Immunofluorescence × 200). **D**: statistic data. *p < 0.05, compared to control; ^#^p < 0.05, compared to *H. pylori*.

Immunofluorescence showed oxidative DNA damage in GES-1 cells after co-culture with *H. pylori*^*30*^. Fluorescent expression was strong in the nucleus, cytoplasm and cell membrane. The expression level of 8-OHdG was significantly increased in GES-1 cells infected with *H. pylori* and *H. pylori*^*30*^. In addition, we also found that the expression of 8-OHdG in GES-1 cells cultured with *H. pylori*^*30*^ was significantly decreased when compared with GES-1 cells cultured with *H. pylori* (Figure 2A-D, P < 0.05).

### Changes in the mitochondrial membrane potential of GES-1 infected with *H. pylori*^*30*^

**Figure 3:**
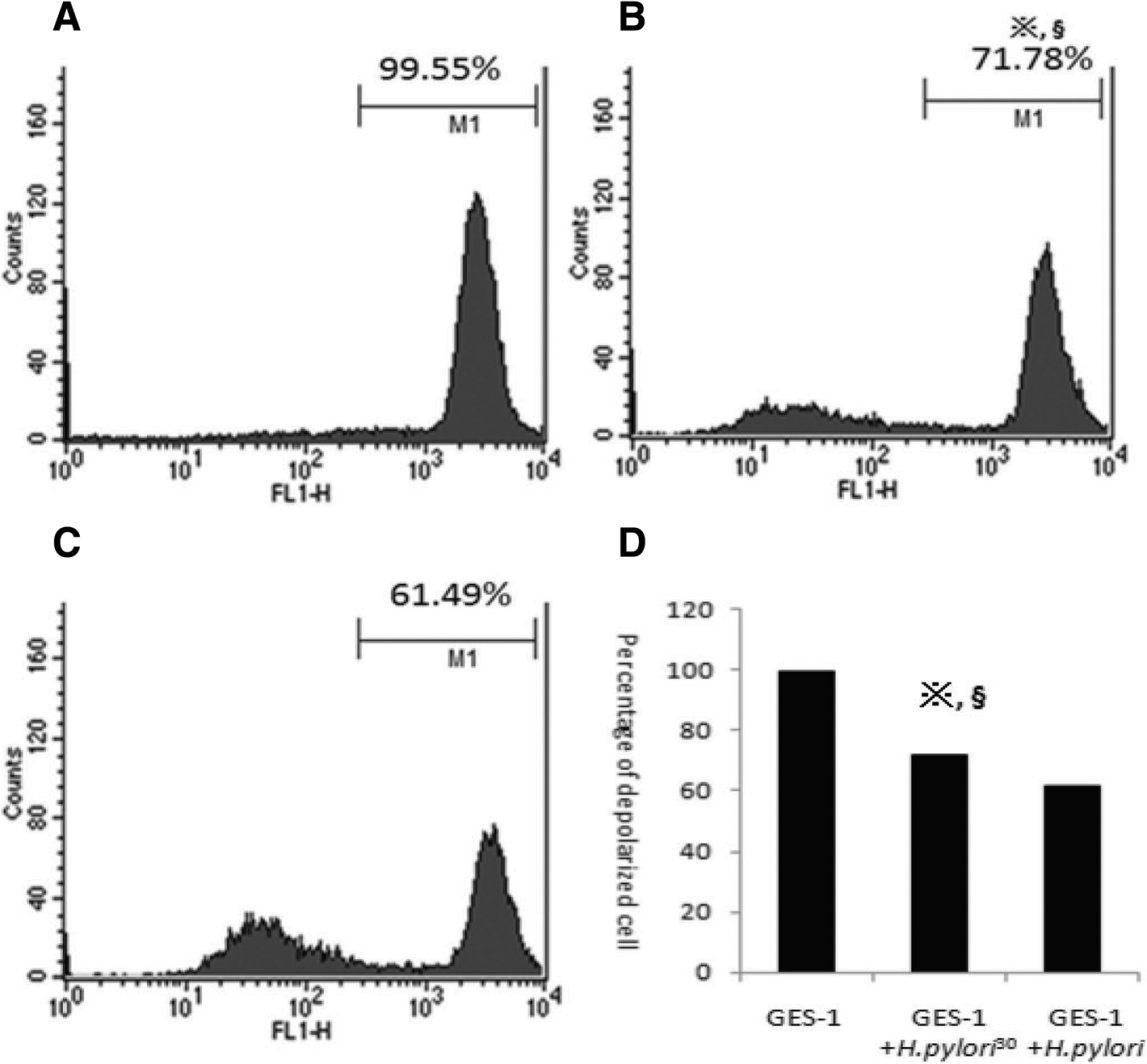
**Changes in mitochondrial membrane potential (ΔΨm). A**: GES-1 cells (negative control group); **B**: GES-1 cells co-cultured with *H. pylori* ^*30*^; **C**: GES-1 cells co-cultured with *H. pylori.* **D**: statistic data. *p > 0.05, compared to control; ^§^ *p* > 0.05, compared to *H. pylori*.

The results showed that GES-1 cells co-cultured with *H. pylori*^*30*^ were not depolarized; however, the cell population shifted to left, indicating no apparent disruption of the mitochondrial membrane potential (*ΔΨ*m). No significant change was detected in mitochondrial membrane potential of GES-1 cells infected with *H. pylori*^*30*^ by flow cytometry, compared with that of the GES-1 control groups (Figure 3A-C, p > 0.05).

### NaCl pretreatment attenuated *H. pylori*-induced apoptosis

**Figure 4:**
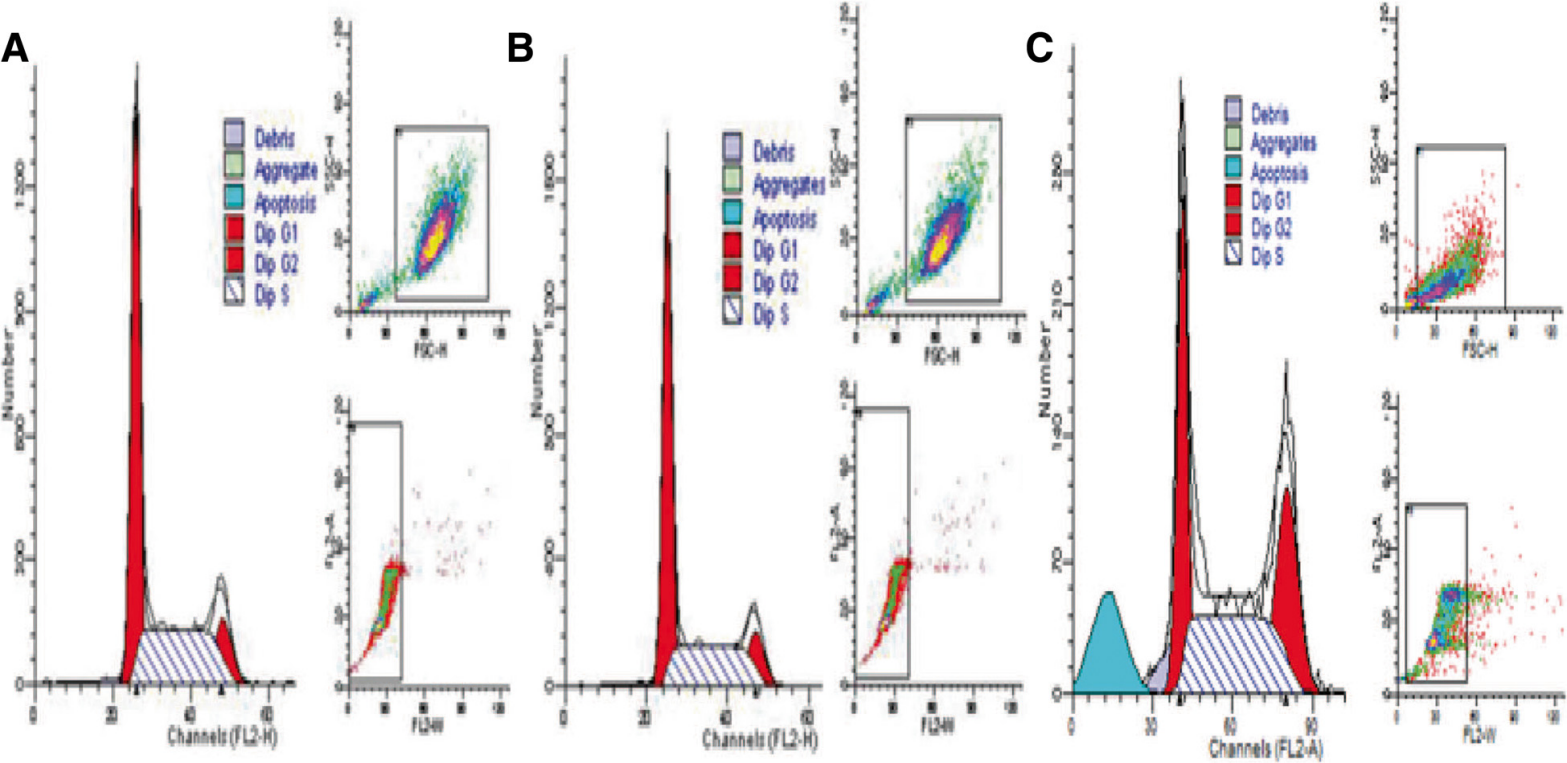
**Cell cycle and apoptosis analysis by flow cytometry.** Cell numbers were calculated according to DNA content of G0/G1, S, and G2/M phases. **A**: negative control GES-1 cells; **B**: GES-1 cells co-cultured with *H. pylori* ^*30*^; **C**: GES-1 cells co-cultured with *H. pylori* (PI staining).

After co-culturing with *H. pylori*^*30*^, flow cytometry showed that apoptotic rate was not significantly increased in GES-1 cells infected with *H. pylori*^*30*^ compared with GES-1 cells. However, the apoptotic rate was increased in GES-1 cells infected with *H. pylori* compared with GES-1 cells infected with *H. pylori*^*30*^ (Figure 4A-C, p > 0.05).

**Figure 5:**
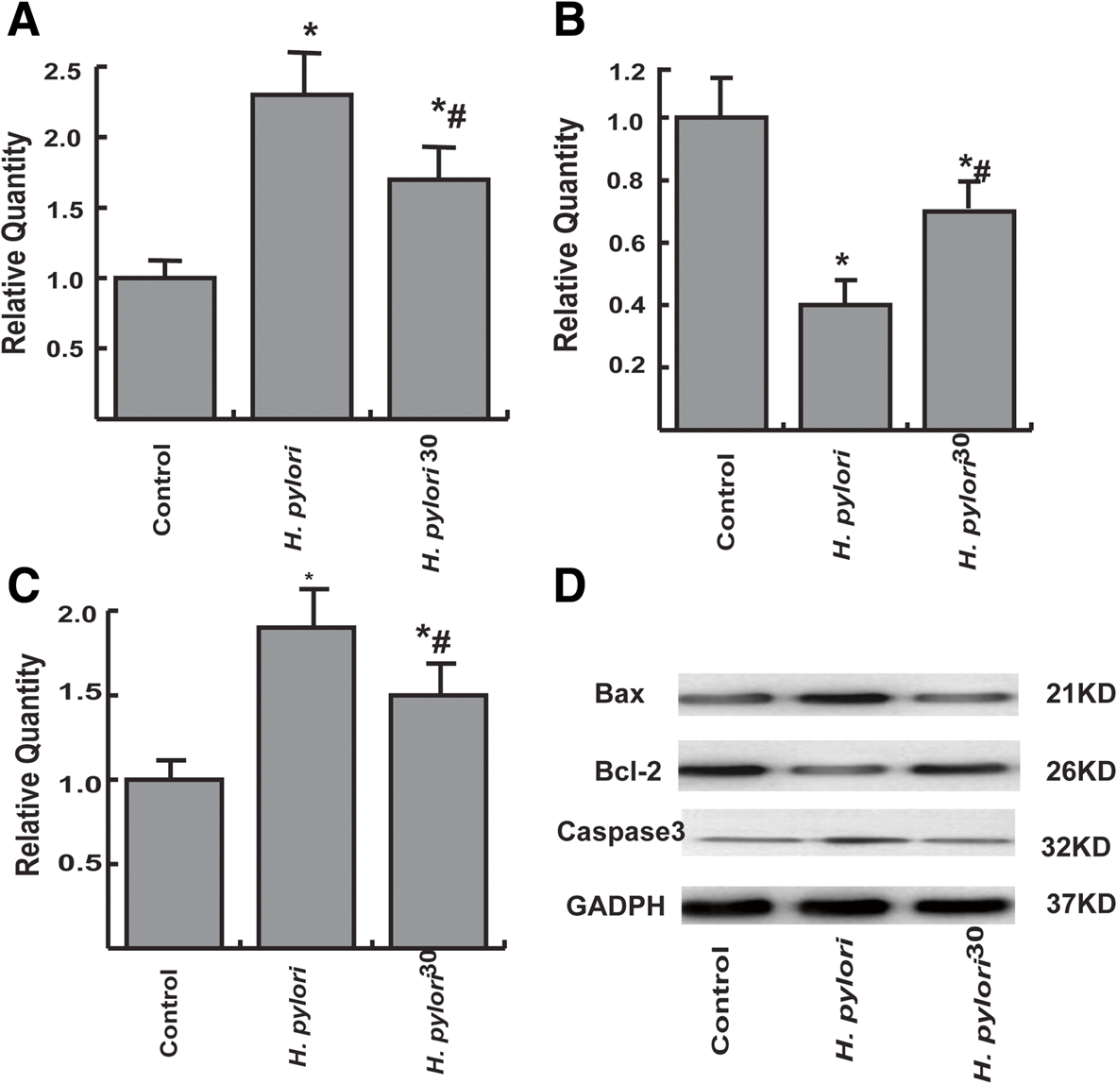
**Protein markers of apoptosis detection by Western blot.** Western blots were performed to detect the abundance of apoptosis related proteins. **A**: Bax; **B**: Bcl-2; **C**: Caspase3. All proteins were normalized to the corresponding β-actin. **D**: western blot image. *p < 0.05, compared to control; ^#^p < 0.05, compared to *H. pylori*.

To analyze further the effect of high salt pretreatment on *H. pylori*-induced apoptosis, we also detected established protein markers of apoptosis including Bax, Bcl-2 and Caspase3 proteins using Western blot. The results suggested that there were significant increased in Bax and Caspase3 proteins and decreased in Bcl-2 protein in GES-1 cells infected with *H. pylori* and *H. pylori*^*30*^. However, there were significant decreased in Bax and Caspase3 proteins and increased in Bcl-2 protein in GES-1 cells infected with *H. pylori*^30^ when compared with GES-1 cells cultured with *H. pylori.* These results demonstrated that high salt pretreatment attenuated *H. pylori*-induced apoptosis (Figure 5A-D, P < 0.05).

### NaCl pretreatment exacerbated *H. pylori*-induced proliferation of gastric epithelial cells

**Figure 6:**
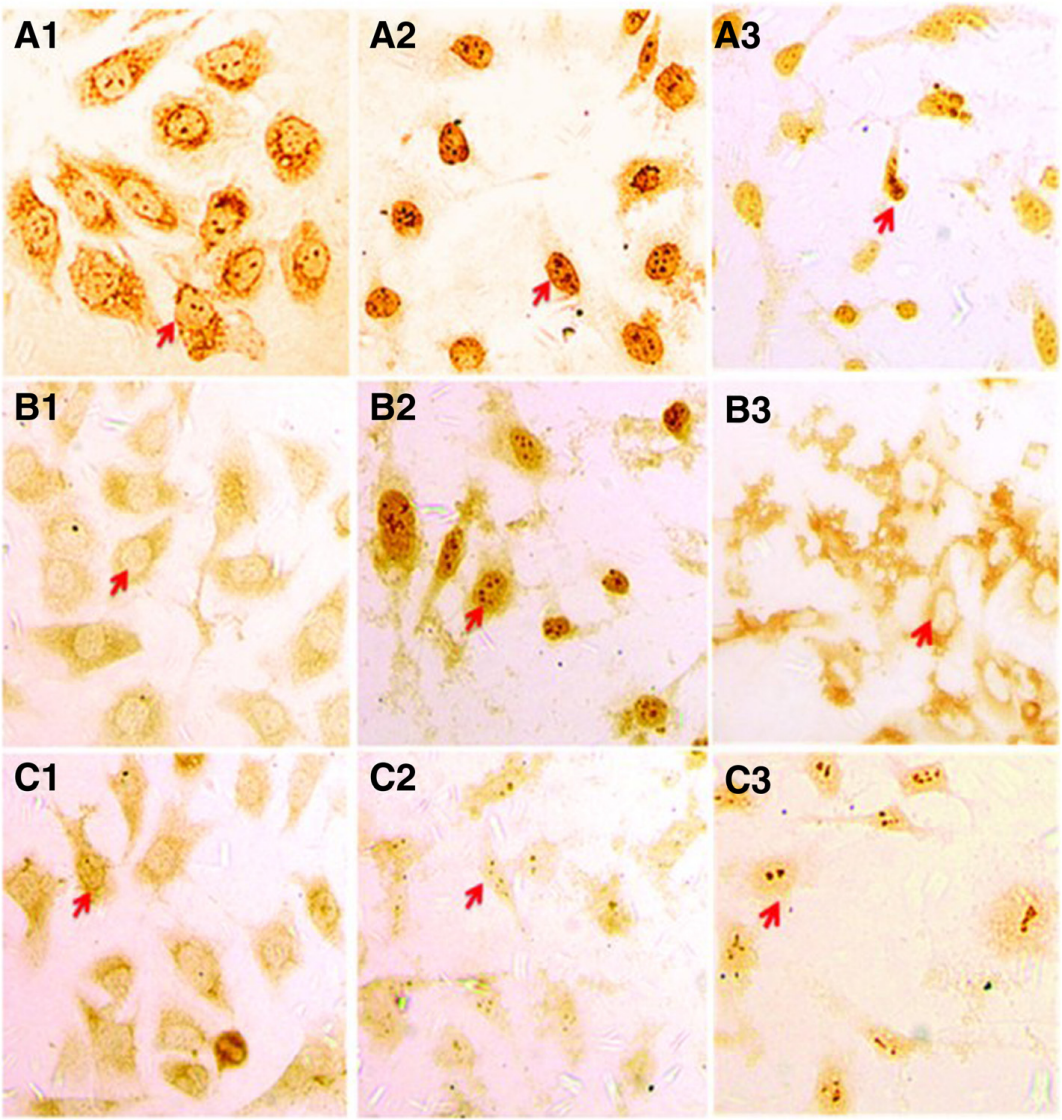
**Expression of Ki-67, PCNA and P21 protein in GES-1 cells. A**: Ki67 antigen was mostly located in the nuclei of GES-1 cells. The intensity of Ki67 was higher in the *H. pylori* ^*30*^ group (A2) than in the control group (A1) and *H.pylori* group (A3). **B**: PCNA antigen was mostly located in the nuclei of GES-1 cells; The intensity of PCNA was higher in the *H. pylori* ^*30*^ group (B2) than in the control group (B1) and *H. pylori* group (B3). **(C)** Expression of P21 was mostly located in the cytoplasm of GES-1 cells; the expression intensity of P21 was lower in the *H. pylori* ^*30*^ group (C2) than in the control group (C1) and *H. pylori* group (C3) (Immunohistochemical staining × 200).

Immunohistochemical staining showed that after co-culturing with *H. pylori*^*30*^, the expression of Ki-67 and PCNA was significantly increased in GES-1 cells (p < 0.05), compared with that in control cells. The expression of Ki-67 and PCNA was mostly located to the nuclei of experimental GES-1 cells compared with the control groups (Figure 6A-B). The expression levels of p21 was significantly decreased in GES-1 cells compared to negative controls. The expression of p21 was mostly located to the cytoplasm in the GES-1 cells after co-culture with *H. pylori*^*30*^ (Figure 6C)*.*

## Discussion

*H. pylori* was found to survive and adapt to high salt stress through alterations in biological characteristics of viability, morphology and expression of virulence factors. Besides, high salt (NaCl) diet is closely associated with high incidence of gastric illness [14,15]. The risk of gastric cancer in high salt diet population is significantly higher than that in low salt diet population [16]. The previous experiments showed that high salt solution resulted in gastric mucosal hyperemia, erosion and ulceration in rats [17]. High salt diet is found to be closely linked with *H. pylori* infection, which causes gastric mucosal injury and temporarily damages mucous barrier [18]. Too much salt intake increases *H. pylori* colonization in gastric mucosa and promotes mucosa malignant transformation [19]. The synergy of *H. pylori* and 10% high salt diet induces gastric mucosa to express inflammatory cytokines iNOS and Cox-2 and aggravates mucosal damage in MGs [20]. High salt has been widely used to kill microbes in preservation of foods because of the difference in osmolality between the outside and inside of bacterial membrane. In the present study, we initially observed the morphological changes of GES-1 cells after co-culturing with *H. pylori* that had been pretreated with high concentration NaCl. The GES-1 cells morphologically transformed from multiangular to round or irregular shapes, and occurred in various sizes, with disrupted cell walls, cytoplasmic vacuoles and cytoplasmic leakage. These findings suggested that *H. pylori*^*30*^ retained the ability to damage GES-1 cells.

*H. pylori* could stimulate gastric epithelial cells to generate a large number of reactive oxygen species (ROS), the accumulation of which can cause oxidative damage to lipids, proteins and DNA [21]. 8-OHdG is a product of oxidative DNA damage and serves as an established marker of oxidative stress related to carcinogenesis [22]. In the current study, we found that the expression level of 8-OHdG was significantly increased in GES-1 cells infected with *H. pylori* and *H. pylori*^*30*^. In addition, we also found that the expression of 8-OHdG in GES-1 cells cultured with *H. pylori*^*30*^ was significantly decreased when compared with GES-1 cells cultured with *H. pylori.* The results suggested that high salt pretreatment attenuated *H. pylori*- induced oxidative DNA damage.

Cell proliferation and apoptosis are essential events in the cellular turnover of gastric tissue. Previously published reports have suggested that mucosal oxidative damage due to *H. pylori* infection was associated with increased inflammatory cell infiltration, enhanced apoptosis and cell proliferation [23]. Although no significant disruption of the mitochondrial membrane potential (*ΔΨ*m) and apoptotic rate were observed compared with control groups in this study, there were significant decresed in Bax and Caspase3 proteins and increase in Bcl-2 protein in GES-1 cells infected with *H. pylori*^30^ when compared with GES-1 cells cultured with *H. pylori.* These results indicated that NaCl pretreatment attenuated *H. pylori*-induced apoptosis. Previous studies generally thought that bacteria might not be able to survive in 30% of saturated sodium chloride solution, but Loh et al. [24] found when *H. pylori* was cultured with various concentrations (0.25% through 2%) of salt in media, the *H. pylori* 26695 *cagA* transcription and expression increased in a salt concentration-dependent manner. Rogers et al. [25] found that after high salt pretreatment, G27 *H. pylori* strains gradually stopped reproduction, and ultimately died. The results suggested there was *H. pylori* strain heterogeneity for tolerance to high salt environment. Some *H. pylori* strains were more fragile to high salt environment. Our previous findings [26] showed that 30% of sodium chloride induced the biologically characteristic alterations of *H. pylori* with deceased ATP level and reduced virulence gene transcription and expression, but with increased urease activity and cellular colonizing potential. Thus, we are considered that NaCl pretreatment attenuated *H. pylori*-induced apoptosis might be associated with the reduced virulence of *H. pylori.*

Ki67 is a nuclear proliferation-associated antigen, the expression of which is strictly associated with cell proliferation. It is present during all active phases of the cell cycle (G1, S, G2, and mitosis), but is absent from resting cells (G0). Ki67 protein expression also has a positive correlation with the activity of tumor proliferation and prognosis; therefore, it is considered to be a reliable cellular marker to determine the growth fraction of a given cell population [27]. Our study found that the expression of Ki-67 in GES-1 cells was significantly increased after co-culturing with *H. pylori*^*30*^. Proliferating cell nuclear antigen (PCNA), a known cofactor of DNA polymeraseδ, is closely associated with cell proliferation [28]. PCNA forms a homotrimer that circles the DNA and operates as a scaffold to assemble a multitude of proteins required for DNA unwinding and synthesis, cell cycle progression and chromatin structure maintenance [29]. The p21 protein exists in a quaternary complex with a cyclin, a cyclin-dependent protein kinase (CDK) and PCNA. The p21 protein has been found to mediate p53-induced growth arrest triggered by DNA damage by inhibiting p53 activation to decrease cell proliferation [30]. The p21 protein blocks the initiation of DNA-replication by inhibiting CDK complexes [31] and blocks the action of PCNA. In the present study, we found that NaCl-pretreated *H. pylori* can specifically affect the proliferation of GES-1 cells, while increasing PCNA expression and decreasing P21 expression, which suggested that *H. pylori*^*30*^ may enhance cell proliferation through the PCNA/P21 signal pathway.

The DNA oxidative damage and increased proliferation of GES-1 cells observed in our study may be due to the release of CagA or other virulence factors from *H. pylori*, which stimulated the corresponding receptors in GES-1 and led to the production of inflammatory mediators and the formation of oxygen free radicals and consequently promoted cell proliferation. Virulence factor CagA and urease can induce cell proliferation. Yan et al. found that CagA+ *H. pylori* culture filtrates induced DNA damage in human gastric epithelial cells *in vitro*, and accelerated human gastric epithelial cell proliferation and altered their morphology [32]. In our preliminary study, the level of CagA protein increased with NaCl concentrations. Abnormal regulation of *H. pylori* and CagA expression in response to high salt concentrations may be a potential factor in the development of gastric carcinoma although the molecular mechanisms remain unclear.

## Conclusion

In conclusion, GES-1 cells co-culturing with NaCl-pretreated *H. pylori* exhibited oxidative DNA damage accompanied with morphological alterations and cell proliferation. Our study provided *in vitro* evidence that *H. pylori* can adapt to high salt environmental stress and delay pathogenecity, which may facilitate its transmission and infection in gastric carcinogensis.

## Materials and methods

### Culture and pretreatment of *H. pylori* with high-salt

*H. pylori* (L301 strains), which is positive for CagA and VacA, was kindly provided by the Third Laboratory of Cancer Institute, China Medical University. The bacteria were grown on brain heart infusion agar supplemented with 7% sheep blood, 0.4% BBLTM IsoVitaleXTM Enrichment, 0.08% amphotericin B, 0.2% vancomycin and 0.5% trimethoprim (Sigma,USA), and incubated under microaerophilic conditions at 37°C and 95% humidity. Media routinely used for *H.pylori* culture contain physiological concentrations of NaCl. In our study, the media used for the experimental group were supplemented with 30% NaCl (*H.pylori*^*30*^) [26].

### Co-culture of GES-1 with *H. pylori*

Co-culture of GES-1 with *H. pylori* was done as described previously [33]. Briefly, GES-1 was grown in RPMI 1640 media containing 10% fetal bovine serum, 100 U/ml penicillin, and 100 μg/ml streptomycin at 37°C in 5% CO_2_. *H. pylori* cells in the exponential phase were added to GES-1 cells (200:1 for *H. pylori* to GES-1 cells) for 24 h. The experimental group was a co-culture of GES-1 and *H. Pylori* cells, whereas GES-1 cells co-cultured with or without *H. pylori* was separately defined as a positive control group and negative control group, respectively.

### Cell morphology

The morphology of GES-1 cells from the three groups co-cultured with or without *H. pylori* was observed by light microscopy. GES-1 cells were placed on chamber slides, fixed with acetone at 4°C and stained with hematoxylin and eosin (HE) for morphologic observation. The ultrastructure of GES-1 cells was observed by transmission electron microscopy (H-600, Olympus, Japan).

### Oxidative DNA damage in GES-1 cells detected by immunofluorescence

Immunofluorescence was done as described previously [34]. Briefly, GES-1 cells were washed twice with PBS and fixed on slides with acetone at 4°C. The slides were incubated in bovine serum albumin for 30 min and with mouse anti-8-OHdG antibody (1:20, Santa Cruz, USA) overnight at 4°C, and then incubated with goat anti mouse IgG antibodies (Santa Cruz, USA) at 37°C for 1 h. The cells were observed by upright fluorescence microscopy. The total gray value average was obtained by measuring five randomly selected fields per slide with a micro ELISA reader (Bio-Tek, USA).

### Alterations in mitochondrial membrane potential of GES-1 cells detected by flow cytometry

The fluorescent dye rhodamine 123 was used to assess changes in mitochondrial membrane potential following co-culture with *H. pylori*. Briefly, 1x10^6^ cells were incubated with 2×10^8^*H. pylori*^*30*^ or *H. pylori* cells for 24 h. The cells were resuspended, washed in PBS twice, and incubated with 5 μM rhodamine 123 at 37°C for 30 min. The cells were then analyzed using a FACStar flow cytometer (BD, USA) and alterations in mitochondrial membrane potential were quantified using ModFit software [35].

### Cell cycle and apoptosis rate detection by flow cytometry

The apoptosis rate was detected by flow cytometry. Briefly, after co-culturing with *H. pylori* or *H. pylori* for 24 h, 1 × 10^6^ GES-1 cells were ingested by trypsin and washed with PBS (pH 7.3-7.4) three times, then fixed with 70% ethanol. After incubation for 20 min, cells were stained with 50 mg/L propidium iodide.

### Protein markers of apoptosis detection by Western blot

Western blotting was done as described previously [36]. Briefly, approximately 20 μg (20 μL) protein per gel well was loaded and resolved by 10% sodium dodecyl sulfate polyacrylamide gel electrophoresis (SDS-PAGE). The SDS-PAGE gel was transferred to polyvinylidene difluoride membranes. The membranes were incubated in tris-buffered saline (TBS) containing 3% non-fat dry milk and a specific proportion of the primary antibodies for Bax, Bcl-2 and Caspase3 proteins(Santa cruz, USA) overnight at 4°C. The blot was then washed and incubated with goat anti-mouse IgG conjugated to peroxidase (Santa cruz, USA). Antibody binding was detected by chemoluminescence staining using the ECL detection kit (Bio-Rad). The density of each band was quantified by densitometry of Bandscan 5.0 software.

### Immunocytochemistry assay for protein expression of biomarkers related to cell proliferation

Expression of proliferation-related proteins was evaluated using the immunocytochemical staining [37]. In brief, 5x10^5^ GES-1 cells were cultured on glass cover slips overnight, and co-cultured with 1×10^8^*H. pylori* or *H. pylori* cells for 24 h. The cells were fixed with 4% paraformaldehyde in PBS for 15 min followed by permeabilization with 0.5% Triton X-100 in PBS for 3 min. The cells were incubated with mouse anti-Ki 67, anti-PCNA or P21 antibody(Santa cruz, USA) at 4°C overnight. The cells were washed three times with PBS and incubated with biotinylated rabbit anti-mouse IgG secondary antibody for 30 min. A positive staining signal was developed by incubating the cells in 3,3-diaminobenzidine (DAB) solution for 10 min at RT in the dark. Cells from five randomly selected fields were immunostained on cover slips and microscopically reviewed.

### Statistical analysis

Statistical analysis was performed using SPSS version 18.0 software. Results were expressed as the mean ± standard deviation and compared between groups using the Student t-test. A *p* value < 0.05 (two-sided) was considered statistically significant.
